# Understanding Local Reactions Induced by *Bothrops jararaca* Venom: The Role of Inflammatory Mediators in Leukocyte–Endothelium Interactions

**DOI:** 10.3390/biomedicines12040734

**Published:** 2024-03-26

**Authors:** Bianca Cestari Zychar, Luís Roberto C. Gonçalves

**Affiliations:** Laboratório de Fisiopatologia, Instituto Butantan, Av. Vital Brazil 1500, São Paulo 05503-900, Brazil

**Keywords:** *Bothrops jararaca*, snakebite, local lesions, inflammation, leukocyte–endothelium interactions, antivenom, anti-inflammatory drugs, microcirculation

## Abstract

In recent years, extensive research has delved into the pathophysiology of local reactions triggered by *Bothrops* snake venoms. Even though antivenom works well at reducing death and systemic effects, it is still not very effective in treating local reactions because it cannot counteract damage that has already been triggered. This limitation might be attributed to certain molecules that amplify the venom-induced innate response. While evidence suggests endogenous mediators at the venom site play a role in this envenomation, in Brazil, the concurrent use of anti-inflammatory agents or other drugs alongside antivenom remains uncommon. This study evaluated the pharmacological mediation of alterations in leukocyte–endothelium interactions following the experimental envenomation of mice with *Bothrops jararaca* venom, the main culprit of snake-related accidents in Southeast Brazil. We treated envenomed mice with inhibitors of different pharmacological pathways and observed the cremaster muscle microcirculation with intravital microscopy. We found that eicosanoids related to cyclooxygenase pathways and nitric oxide significantly contributed to *B. jararaca* venom-induced alterations in leukocyte–endothelium interactions. Conversely, lipoxygenase-mediated eicosanoids, histamine, and serotonin had minimal participation. Notably, dexamethasone and antivenom treatment diminished *B. jararaca* venom–induced alterations in leukocyte–endothelium interactions. The limited efficacy of the antivenom in managing *Bothrops* venom-induced local reactions emphasizes the critical need for supplementary treatments to enhance therapeutic outcomes.

## 1. Introduction

Snakebites are a serious global health issue; they are especially prevalent among underprivileged populations in tropical and subtropical areas and lead to significant social problems [1,2,3]. The World Health Organization (WHO) estimates that throughout the world, there are approximately 2.7 million snakebite incidents each year, resulting in about 81,000 to 138,000 fatalities and 400,000 survivors with enduring disabilities [4,5]. According to the WHO, snakebites are a neglected tropical disease. Indeed, since 2017, snakebite envenomation has been listed among the 20 neglected tropical diseases, prompting a strategic plan to reduce fatalities by 50% by 2030. Despite being preventable and treatable, research investments in this area remain remarkably low [6]. The Brazilian Academy of Sciences has identified accidents involving venomous animals as a neglected disease in Brazil and prioritizes support for research on drug combinations to treat toxin-induced accidents [7].

In Brazil, an average of 29,000 snakebite cases occur each year, resulting in approximately 120 fatalities and around 600 cases with curable sequelae, excluding unreported cases. Among venomous snakes, the Viperidae family, particularly the subfamilies Crotalinae (including snakes of the genera *Crotalus*, *Bothrops*, and *Lachesis*), are noteworthy [8,9]. *Bothrops* snakebites cause systemic reactions, including severe blood clotting disorders [10,11] such as disseminated intravascular coagulation [12]. Complications such as hypotension and hypovolemic shock can lead to fatalities. Severe local reactions comprising edema, pain, hemorrhage, and necrosis are common, often resulting in substantial tissue loss and potential limb amputations [13,14]. These reactions trigger inflammatory responses by activating signaling pathways that cause the transcription of inflammatory genes, such as cytokines and eicosanoids [15]. The increased production of these factors leads to endothelial activation with the expression of lectins and adhesion molecules, resulting in leukocyte-endothelium interactions, including firm adhesion and cell migration [16]. The pathogenesis of local reactions caused by *Bothrops* venoms is still not fully understood because they are made up of many different toxins that can work alone or together through various pathways in the body [17,18].

The only effective treatment for snakebite envenomation is specific antivenom [6,19,20]. Despite its efficacy in reducing lethality and reversing systemic effects, antivenom inadequately addresses local reactions, which cause severe sequelae [10,11,13]. This deficiency is due to the rapid onset of these local actions and the fact that antivenom cannot reverse established or triggered damage or neutralize endogenous mediators involved in the process [10,21].

Essential anti-inflammatory drugs include glucocorticoids (steroids), which inhibit early and late inflammatory manifestations, and non-steroidal drugs, which are widely used throughout the world [22]. The literature has shown that treating animals with anti-inflammatory agents, particularly phospholipase A_2_ and cyclooxygenase (COX) inhibitors, significantly reduces edema induced by *Bothrops* venoms [23,24,25]. Despite evidence showing that endogenous mediators are released at the venom injection site, the concurrent use of anti-inflammatory agents or other drugs alongside serotherapy remains uncommon in Brazil [14]. The limited effectiveness of serotherapy in treating *Bothrops* venom-induced local reactions has prompted the search for more efficacious complementary treatments. In recent decades, many researchers have sought to understand local reactions caused by *Bothrops* venoms and to explore complementary therapies that could be given alongside antivenom. Improving antivenoms so that they are more efficacious [26], using protease inhibitors [27], identifying animals resistant to snake venom [28], and utilizing therapeutic combinations, including anti-inflammatory drugs that work with serotherapy [25,29], are a few of these endeavors.

The present study aimed to ascertain which inflammatory mediators alter leukocyte–endothelium interactions following experimental *Bothrops jararaca* envenomation in mice. The goal was to find possible treatments that could be used alongside antivenom to help reverse the severe, early effects of the venom. The results demonstrated that alterations in the leukocyte–endothelium interactions induced by *Bothrops jararaca* venom (BjV) are mediated mainly by COX-derived eicosanoids and nitric oxide (NO). Moreover, treatment with dexamethasone and antivenom could reduce the alterations in leukocyte-endothelium interactions.

## 2. Materials and Methods

### 2.1. Animals

Male Swiss mice weighing 20–25 g supplied by the housing facility of the Instituto Butantan were used. Before the experiments, the mice were maintained for 2 days on a 12 h photoperiod and received water and food ad libitum. All experimental procedures were conducted according to the ethical parameters proposed by the International Society of Toxinology by the Brazilian College of Experimental Animals and were approved by the Ethical Committee for the Use of Animals of Butantan Institute (protocol n° 199/05).

### 2.2. BjV

The Laboratory of Herpetology at the Butantan Institute provided a pool of lyophilized venom obtained from various adult *B. jararaca* specimens. The venom was stored at −20 °C. Solutions (*w*/*v*) were prepared immediately before use in sterile saline.

### 2.3. Antivenom

The Butantan Institute produced the *Bothrops* antivenom (BAV) that was kept at 8 °C until use. BAV is produced by immunizing horses with a combination of antigens from *B. jararaca*, *Bothrops jararacussu*, *Bothrops moojeni*, *Bothrops neuwiedi*, and *Bothrops alternatus*. The antivenom is subjected to multiple treatments during production to obtain the F(ab’)_2_ fraction of IgG. BAV is available in 10 mL vials, and according to the supplier, each 1 mL of BAV neutralizes 5 mg of the reference BjV.

### 2.4. Treatment of Mice

The mice were divided into groups (*n* = 5 per group) and received the following treatments 30 min or 1 h before or after a subcutaneous injection of BjV into the scrotal bag.

Group 1: Dexamethasone (a corticosteroid; Decadron, Aché, São Paulo, SP, Brazil) was injected intraperitoneally (1 mg/kg, dissolved in sterile saline) 1 h before or after the subcutaneous injection of BjV [25].

Group 2: Indomethacin (a COX inhibitor; Sigma, Livonia, MI, USA) was injected intraperitoneally (3 mg/kg, dissolved in 1 M Tris buffer, pH 8, at 37 °C and sterile saline solution [1:10]) 30 min before the subcutaneous injection of BjV [25].

Group 3: Celecoxib (a COX2 inhibitor; Celebra^®^, Pfizer, Itapevi, SP, Brazil) was administered orally (30 mg/kg) 30 min before the subcutaneous injection of BjV. Each Celebra^®^ tablet containing 200 mg of celecoxib was dissolved in 2.5 mL of 1% carboxymethyl (CMC) solution. This solution was used for treatment [30].

Group 4: Nordihydroguaiaretic acid (NDGA, a 5-lipoxygenase inhibitor; Sigma, Sigma, Livonia, MI, USA) was administered intraperitoneally (30 mg/kg) 30 min before the subcutaneous injection of BjV. For every 30 mg/kg of the compound, 2 mL of ethanol–saline (1:9, *v*/*v*) was added, and the pH was adjusted to 7.5 with 0.1 N NaOH [31].

Group 5: L-NG-nitro arginine methyl ester (L-NAME, a nonspecific inhibitor of nitric oxide synthase [NOS]; Sigma, Livonia, MI, USA) was injected subcutaneously (100 mg/kg, dissolved in sterile saline) 30 min before the subcutaneous injection of BjV [32].

Group 6: Aminoguanidine hydrochloride (a selective inducible nitric oxide [iNOS] inhibitor; Sigma, Livonia, MI, USA) was injected intraperitoneally (50 mg/kg, dissolved in sterile saline) 30 min before the subcutaneous injection of BjV [32].

Group 7: Promethazine hydrochloride (an H1 receptor inhibitor; Sigma, Livonia, MI, USA) was injected intraperitoneally (10 mg/kg, dissolved in sterile saline) 30 min before the subcutaneous injection of BjV [31].

Group 8: Methysergide maleate salt (a serotonin 5-HT receptor antagonist; Sigma, Livonia, MI, USA) was injected intraperitoneally (0.8 mg/kg, dissolved in sterile saline) 30 min before the subcutaneous injection of BjV [31].

Group 9: BAV was injected intravenously (0.2 mL) alone or with dexamethasone 1 h after the subcutaneous injection of BjV. The control group received saline rather than a drug by the same routes [25].

### 2.5. Intravital Microscopy of Murine Cremaster Venules

Intravital microscopy was used to evaluate leukocyte–endothelial interactions in mouse cremaster muscle venules. Five mice per group were injected under the skin of their scrotum with either 100 µL of sterile saline (as a control) or 1 µg of BjV (in a total volume of 100 µL). This BjV dose is sub-hemorrhagic, as determined in a previous study [26]. After 2 or 24 h, the mice were anesthetized with an intraperitoneal injection of ketamine (100 mg/kg) and xylazine (10 mg/kg), and the cremaster muscle was exteriorized for microscopic examination in situ as described by Baez [33]. The microcirculation of the cremaster muscle was visualized in a transparent board window heated at 37 °C, on which the anesthetized mouse was maintained. Leukocyte responses in the cremaster muscle microcirculation were evaluated by light microscopy (Axioplan II, Carl Zeiss, Oberkochen, Germany, equipped with Achroplan objectives with 10.0 longitudinal distance, 0.25 numeric aperture, and 1.60 optovar). Images were captured on a computer for further analysis using image analyzer software (KS 300, Kontron, Carl Zeiss, Germany). One post-capillary venule (30–40 mm in diameter) was randomly selected. After the stabilization period (10 min), the number of adhered leukocytes was counted for a period of 5 min in a 100 µm vascular section. Adherent leukocytes were cells that remained immobile for at least 30 s within a given 100 µm vessel segment. The results are expressed as the number of cells per 100 µm. It was also possible to count the number of leukocytes that had migrated to tissue outside of vessels within 50 µm of all sides of the 100 µm vessel segments. The number of adhered leukocytes was evaluated 2 h after BjV envenomation, while the number of migrated leukocytes was evaluated 24 h after BjV envenomation [26].

### 2.6. Statistical Analysis

The results are expressed as the mean ± standard error of the mean (*n* = 5 mice per group) and were analyzed with one-way analysis of variance (ANOVA), followed by Tukey’s test. A *p*-value < 0.05 was considered to be statistically significant. Two independent experiments were performed.

## 3. Results

We evaluated microcirculatory dysfunction with intravital microscopy at 2 and 24 h after BjV envenomation. We assessed changes in leukocyte–endothelium interactions by counting the number of adhered and migrated cells. We selected these time points based on previous studies [26,27]. At the early time point, most leukocyte-endothelium interactions involve cell adhesion. Later, leukocytes migrate to the extravascular space due to adhesion molecules that present sequential and orchestrated expression kinetics [34].

**Figure 1:**
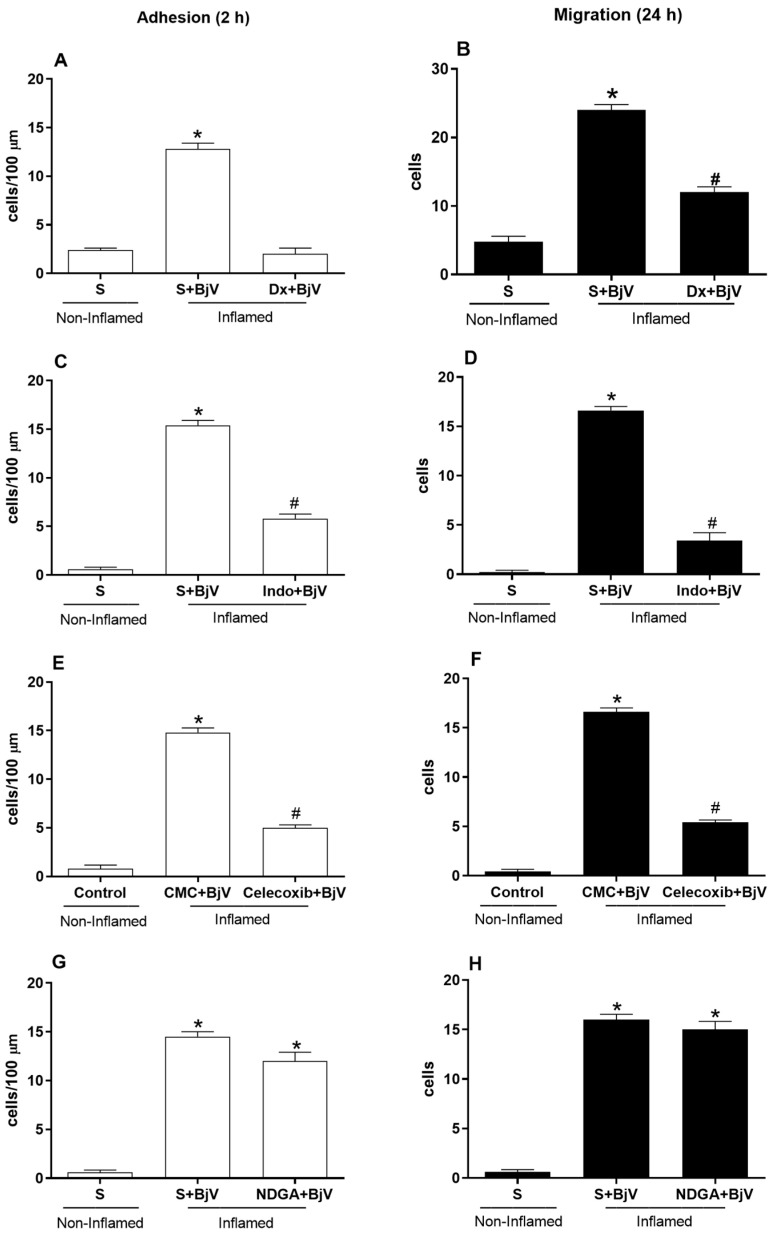
The effect of pretreatment with inhibitors of eicosanoid production on *Bothrops jararaca* venom (BjV)-induced alterations in leukocyte–endothelium interactions in post-capillary venules of the mouse cremaster muscle. The mice were treated with dexamethasone (Dx, 1.0 mg/kg) (**A**,**B**), indomethacin (Indo, 3 mg/kg) (**C**,**D**), celecoxib (30 mg/kg) (**E**,**F**), nordihydroguaiaretic acid (NDGA, 30 mg/kg) (**G**,**H**), saline (S, as a control), or carboxymethyl cellulose (CMC, the control for celecoxib) 30 min or 1 h before subcutaneous injection of BjV (1 μg/100 μL) or S into the scrotum. The number of adhered cells was evaluated 2 h after BjV envenomation (**A**,**C**,**E**,**G**), and the number of migrated cells was evaluated 24 h after BjV envenomation (**B**,**D**,**F**,**H**). The results are presented as the mean ± standard error of the mean (*n* = 5). * *p* < 0.05 compared with the control group; # *p* < 0.05 compared with the control group and the BjV-envenomed group.

BjV induced changes in leukocyte–endothelium interactions in the mouse cremaster muscle microcirculation, with a significant increase in the number of adhered cells after 2 h (Figure 1A,C,E,G) and migrated cells after 24 h (Figure 1B,D,F,H). We treated mice with dexamethasone 1 h before BjV envenomation to assess whether arachidonic acid degradation metabolites are involved in the BjV-induced alterations in leukocyte–endothelium interactions. This pretreatment reduced the number of adhered and migrated cells at 2 and 24 h, respectively, compared with the control group or the BjV-envenomed group (Figure 1A,B).

We also investigated the role of COX in the cellular events in the cremaster muscle microcirculation by treating mice with indomethacin or celecoxib 30 min before BjV envenomation. Both drugs efficiently reduced the number of adhered cells (Figure 1C,E) and migrated cells (Figure 1D,F). However, pretreatment with NDGA, a 5-lipoxygenase inhibitor, could not reduce the number of adhered or migrated cells compared with the BjV-envenomed group (Figure 1G,H).

**Figure 2:**
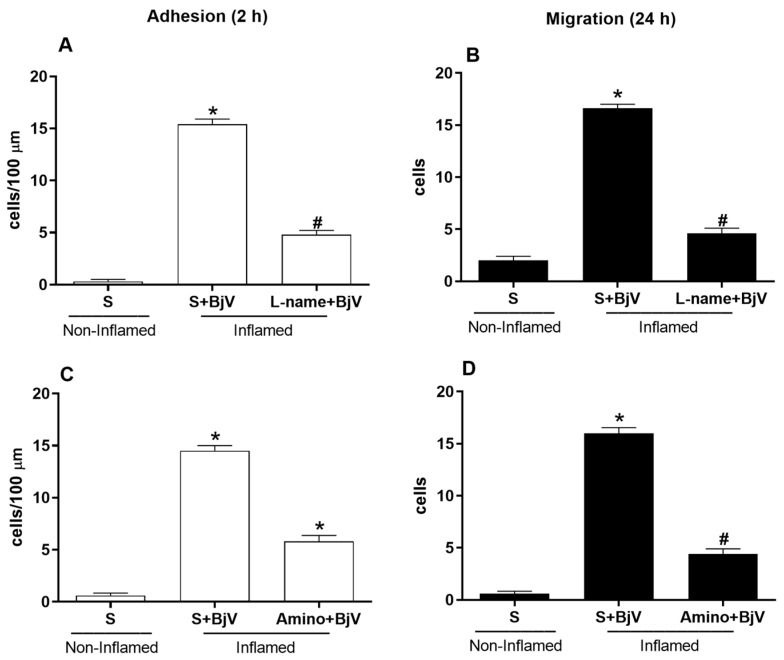
The effect of pretreatment with inhibitors of the nitric oxide pathway on *Bothrops jararaca* venom (BjV)-induced alterations in leukocyte–endothelium interactions in post-capillary venules of the mouse cremaster muscle. The mice were treated with L-NAME (100 mg/kg) (**A**,**B**), aminoguanidine (Amino, 50 mg/kg) (**C**,**D**), or saline (S, as a control) 30 min before subcutaneous injection of BjV (1 μg/100 μL) or S into the scrotum. The number of adhered cells was evaluated 2 h after BjV envenomation (**A**,**C**), and the number of migrated cells was evaluated 24 h after BjV envenomation (**B**,**D**). The results are presented as the mean ± standard error of the mean (*n* = 5). * *p* < 0.05 compared with the control group; # *p* < 0.05 compared with the control group and the BjV-envenomed group.

Other inhibitors that have been found to be effective in preventing microcirculatory changes target the NO pathway. We treated mice with L-NAME, which blocks NOS, or aminoguanidine, which blocks iNOS, 30 min before BjV envenomation. Both inhibitors significantly reduced the number of adhered and migrated cells at 2 and 24 h, respectively (Figure 2).

**Figure 3:**
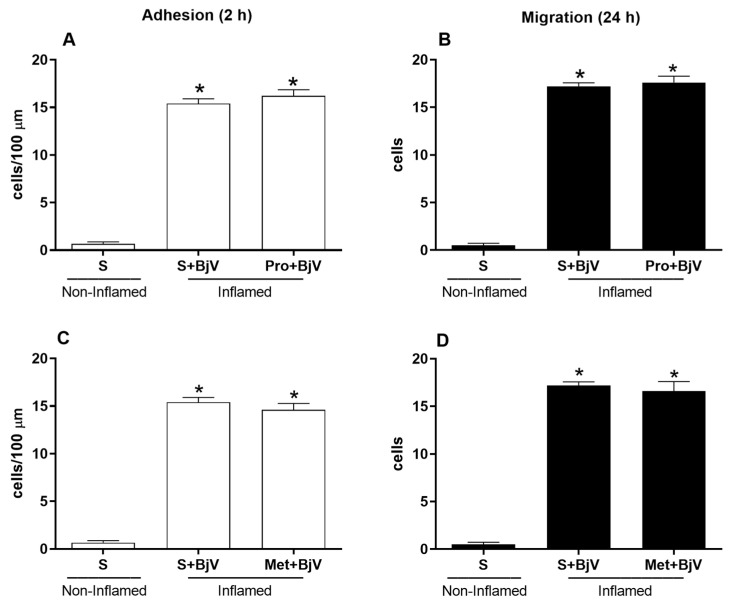
The effect of pretreatment with vasoactive amine inhibitors on *Bothrops jararaca* venom (BjV)-induced alterations in leukocyte-endothelium interactions in post-capillary venules of the mouse cremaster muscle. The mice were treated with promethazine (Pro, 10 mg/kg) (**A**,**B**), methysergide (Met, 0.8 mg/kg) (**C**,**D**), or saline (S, as a control) 30 min before the subcutaneous injection of BjV (1 μg/100 μL) or S into the scrotum. The number of adhered cells was evaluated 2 h after BjV envenomation (**A**,**C**), and the number of migrated cells was evaluated 24 h after BjV envenomation (**B**,**D**). The results are presented as the mean ± standard error of the mean (*n* = 5). * *p* < 0.05 compared with the control group.

Pretreatment with vasoactive amine inhibitors—promethazine, which blocks the histamine H1 receptor, or methysergide, which blocks the serotonin (5-HT2) receptor—did not counteract the BjV-induced alterations in leukocyte–endothelium interactions (Figure 3). None of the drugs used per se caused significant changes in the leukocyte-endothelium interaction patterns.

**Figure 4:**
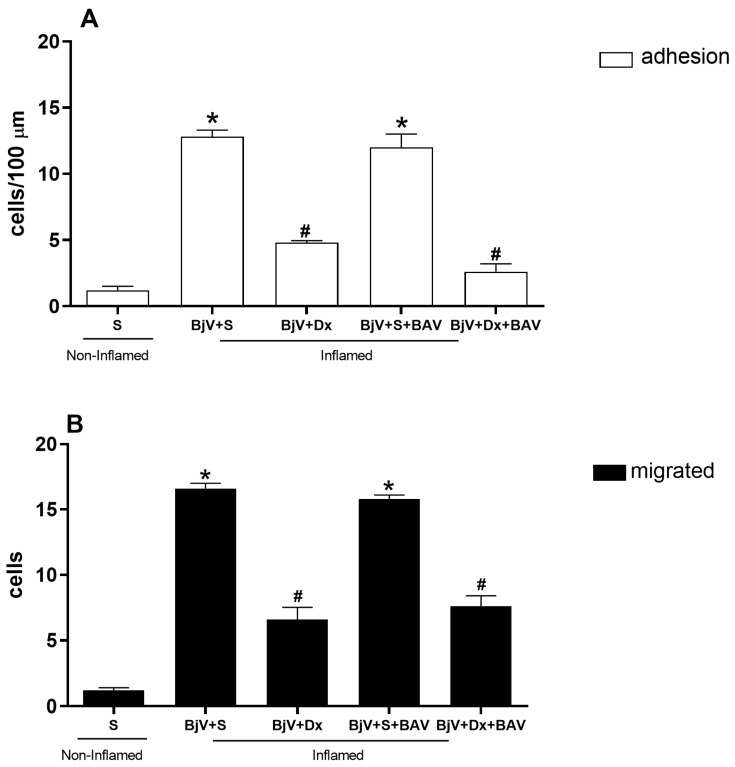
The effect of combined treatment with dexamethasone (Dx) and *Bothrops* antivenom (BAV) on *Bothrops jararaca* venom (BjV)-induced alterations in leukocyte–endothelium interactions in post-capillary venules of the mouse cremaster muscle. The animals were treated with Dx (1.0 mg/kg, intraperitoneal), BAV (200 μL, intravenous), or saline (S, as a control, intravenous or intraperitoneal) 1 h after subcutaneous injection of BjV (1 μg/100 μL) or S into the scrotum. The number of adhered cells was evaluated 2 h after BjV envenomation (**A**), and the number of migrated cells was evaluated 24 h after BjV envenomation (**B**). The results are presented as the mean ± standard error of the mean (*n* = 5). * *p* < 0.05 compared with the control group; # *p* < 0.05 compared with the control group and the BjV-envenomed group.

We also evaluated whether dexamethasone could reverse the inflammatory effects caused by BjV. The administration of dexamethasone 1 h after BjV envenomation decreased the number of adhered cells (Figure 4A) and migrated cells (Figure 4B) compared with the BjV-envenomed group. The administration of BAV 1 h after BjV envenomation could not reduce the number of adhered and migrated cells compared with the BjV-envenomed group (Figure 4A,B). However, treatment with dexamethasone and BAV resulted in fewer adhered and migrated cells (Figure 4A,B). The anti-inflammatory drug dexamethasone appears to reduce the number of leukocytes recruited 1 h after envenomation and thus reduces leukocyte–endothelium interactions. Representative images of adhered and migrated cells in the microcirculation of the cremaster muscle of animals submitted to this set of treatments can be observed in Supplementary Figures S1 and S2, respectively.

## 4. Discussion

According to the WHO, specific antivenoms are the only proven and recommended treatment for snakebites [6]. Since introducing antivenom treatment, there has been a significant decrease in the number of deaths caused by non-medical accidents [14]. Despite the effectiveness of serotherapy in neutralizing systemic symptoms, local reactions induced by *Bothrops* venom do not always respond to such treatment due to the rapid manifestation of these local reactions and the fact that antivenom cannot reverse the already-established or triggered damage or neutralize the endogenous mediators involved in the process [10]. *Bothrops* venoms can induce inflammatory responses, activating signaling pathways that culminate in the transcription of inflammatory cytokines, including tumor necrosis factor alpha (TNF-α), interleukin 1β (IL-1β), and IL-6, and eicosanoids. These molecules lead to the release of vasoactive substances, with a consequent increase in vascular permeability, activation of endothelial cells, and expression of adhesion molecules that trigger leukocyte capture, rolling, firm adherence, and migration [15,34,35,36,37,38].

Previous studies from our group have shown the pro-inflammatory actions of BjV [25,26] due to its metalloproteinases [27]. We have recently shown that the snake venom metalloproteinases Jar, Jar-C, and BnP1 can induce cell adhesion and migration in post-capillary venules in the murine cremaster muscle, as observed by intravital microscopy [39]. Alterations in leukocyte–endothelium interactions occur due to the early participation of intercellular adhesion molecule 1 (ICAM-1) and the later participation of platelet endothelial cell adhesion molecule 1 (PECAM-1) [34].

In the present study, both pre- and post-treatment with dexamethasone could decrease the number of adhered and migrated cells at 2 and 24 h, respectively, after BjV envenomation compared with the control (non-inflamed) and BjV-envenomed (inflamed) groups (Figure 1 and Figure 4). These outcomes demonstrate the participation of arachidonic acid degradation metabolites and the ability of dexamethasone to reverse BjV-induced changes in leukocyte-endothelium interactions. Hence, the eicosanoids originating from COX pathways, but not those originating from the lipoxygenase route, mediate the BjV-induced changes in leukocyte–endothelium interactions. Studies have shown the participation of products originating from eicosanoids in cell migration induced by BjV [40]. Moreover, glucocorticoids reduce cell recruitment to the site of inflammation by inhibiting adhesion molecules, as they induce a rapid change in the surface expression of CAMs via a genomic mechanism, or by interfering with ICAM-1 expression (as observed with IL-1-induced leukocyte adherence in the mouse mesentery) [41,42,43]. Eicosanoids mediate the migration of inflammatory cells induced by *Bothrops* venoms; this recruitment depends on the expression of adhesion molecules such as ICAM-1, leukocyte-endothelial cell adhesion molecule 1 (LECAM-1), lymphocyte function-associated antigen 1 (LFA-1), and PECAM-1, but not macrophage-1 antigen (MAC-1) [40,44,45]. The expression of these adhesion molecules increases leukocyte–endothelium interactions, the first step before inflammatory cell diapedesis [46], which may be reflected in the modifications of cellular events observed after BjV envenomation.

Pretreatment with indomethacin (a COX pathway inhibitor) and celecoxib (a COX-2-specific inhibitor) reinforce the importance of the participation of the COX pathway in mediating the BjV-induced alterations in leukocyte–endothelium interactions. Indeed, these drugs were quite effective in inhibiting the cellular events induced by BjV, with significant reductions in the number of adhered and migrated cells 2 and 24 h after envenomation, respectively, compared with the BjV-envenomed group. These data corroborate the literature, where it has already been shown that the pretreatment of animals with anti-inflammatory drugs and inhibitors of phospholipase A_2_ and the COX pathway significantly reduce paw edema induced by *Bothrops* venoms [23,24,25,31]. Moreover, COX-2 inhibition in mice significantly reduces paw edema [47].

Leukotrienes interact with neutrophil receptors on the leukocyte surface, which leads to chemotaxis and increased adhesion to endothelial cells, mainly through increased expression of β2 integrin [48]. However, pretreatment with NDGA, which blocks the lipoxygenase pathway, did not stop the release of leukotrienes. This suggests that the products of the lipoxygenase pathway are not involved in the effects induced by BjV envenomation. In contrast, products derived from the lipoxygenase pathway participate in cell migration induced by *Bothrops erythromelas* and *Bothrops alternatus* venoms [44].

Research has shown that NO controls leukocyte–endothelium interactions. It is possible to observe more of these cellular events by blocking endothelial nitric oxide synthase (eNOS) and iNOS [32,49]. A study found that nonspecific NOS inhibitors, like L-NAME, do not stop the production of ICAM-1, which helps white blood cells stick firmly to the endothelium when lipopolysaccharide (LPS) or carrageenan is present [50]. However, we found that BjV-induced cellular events in the mouse cremaster muscle microcirculation slowed significantly with L-NAME or aminoguanidine pretreatment. These findings indicate that the NO pathway and its products play a role in the BjV-induced alterations in leukocyte–endothelium interactions. These results are consistent with previous studies. When applied topically to the spermatic fascia of rats, BjV induces changes in leukocyte–endothelium interactions [51]. However, pretreatment with L-NAME reduces the number of rolling and adherent cells [52]. *Bothrops atrox* venom also induces serum NO levels [53]. It is known that cytokines, including interferon gamma (INF-γ) and TNF-α, induce the expression of iNOS and that BjV can induce the release of both these cytokines and NO [54]. Different experimental conditions, administration routes, or inflammatory stimuli can lead to opposing effects on NO expression, suggesting that this mediator often antagonizes the inflammatory response [52,55]. Additional studies with biochemical and molecular analyses of the eicosanoid and NO pathways are needed to prove the pharmacological approach used in this study and could reinforce our findings.

We found that pretreatment with promethazine, a histamine inhibitor, did not counteract the BjV-induced alterations in leukocyte-endothelium interactions. Studies have shown that this compound does not mediate BjV-induced paw edema in mice [28,31]. Of note, in rats, histamine mediates this edema [23]. Pretreatment with methysergide, a serotonin inhibitor, also did not ameliorate BjV-induced alterations in leukocyte–endothelium interactions. Taken together, these results suggest that vasoactive amines are not involved in this microcirculatory process.

Pre- and post-treatment with BAV inhibits BjV-induced edema in mice [25]. However, this inhibition did not occur in the group that received BAV 45 min after the BjV injection. Thus, BAV neutralizes edema formation, but this reduction is more efficient when BAV is administered soon after envenomation [25]. Picolo et al. [56] noted the same result: BAV decreases edema only when administered before injecting BjV into the rat footpad. Some studies suggest that immunoglobulin therapy does not stop local lesions caused by *Bothrops* venoms because it is hard for the immunoglobulin to get to the lesion site [57]. This would justify using only the Fab portion of total IgG to treat snake envenomation [58]. However, there are no significant differences in the reduction in local lesions like edema, hemorrhage, and myonecrosis between animals treated with all three types of serum. This is true whether the antivenom contains total IgG, only the F(ab’)_2_ portion, or even the Fab portion [59,60].

In the present study, dexamethasone could inhibit the BjV-induced alterations in leukocyte–endothelium interactions compared with the control group and the groups that received only BAV. There were also decreases in animals treated with dexamethasone and BAV (Figure 4). When injected directly into a vein without dilution, BAV can increase the interaction between white blood cells and the endothelium. Antivenom contains the preservative phenol, which is primarily responsible for this effect [26]. This association between dexamethasone and BAV accelerates edema regression, reducing muscle damage from *B. jararaca* and *B. jararacussu* venoms [29], and swelling caused by *B. jararaca* venom [25]. When *B. atrox* venom is the cause of tissue damage, the combination of dexamethasone with antivenom is also beneficial, reducing inflammation and accelerating muscle regeneration [21].

A recent study on people who had been envenomed showed that giving anti-inflammatory drugs and antivenom reduced the local effects of snakebites, including inflammatory symptoms [61]. However, treatment with dexamethasone, alone or combined with antivenom, could not prevent or reduce hemorrhagic damage induced by this venom [21,31]. This outcome aligns with the hypothesis that eicosanoids are not involved in the pharmacological mechanisms that mediate local hemorrhage caused by BjV. BjV does not induce the secretion of endogenous corticosteroids or stimulation of the hypothalamic–pituitary–adrenal axis [62]. In this sense, the use of dexamethasone would be essential to avoid inflammatory reactions. However, as this corticosteroid does not alter the local hemorrhagic and coagulant activities of BjV, it cannot be recommended as a replacement for antivenom. When dexamethasone is associated with BAV, it may improve the treatment of BjV envenomation by reducing the inflammatory response (swelling and leukocyte influx). 

## 5. Conclusions

The inefficiency attributed to the antivenom encourages studies of complementary therapies. Hence, the association of anti-inflammatory drugs, such as dexamethasone, with antivenom for treating local inflammatory reactions in *Bothrops* envenomation would be a rational alternative that could be clinically tested. 

## Data Availability

The data presented in this study are available upon request from the corresponding authors.

## Supplementary Materials

The following supporting information can be downloaded at: https://www.mdpi.com/article/10.3390/biomedicines12040734/s1, Figure S1: The panel shows representative photomicrographs of the data of adhered cells of the Figure 4A. Images were obtained in a microscope, Axioplan II, Carl Zeiss, Germany, equipped with Achroplan objectives with 10.0 longitudinal distance, 0.25 numeric aperture, and 1.60 optovar. Red arrows indicate adhered leukocytes. Figure S2: The panel shows representative photomicrographs of the data of migrated cells of the Figure 4B. Images were obtained in a microscope, Axioplan II, Carl Zeiss, Germany, equipped with Achroplan objectives with 10.0 longitudinal distance, 0.25 numeric aperture, and 1.60 optovar. Red arrows indicate migrated leukocytes.
