# Supplementary material for: Understanding Local Reactions Induced by Bothrops jararaca Venom: The Role of Inflammatory Mediators in Leukocyte–Endothelium Interactions
Source: Biomedicines. 2024 Mar 26;12(4):734. doi: 10.3390/biomedicines12040734 (PMC11048348; doi:10.3390/biomedicines12040734)
Supplement: Supplementary file 1 [file biomedicines-12-00734-s001.zip › biomedicines-2819510-supplementary.pdf]

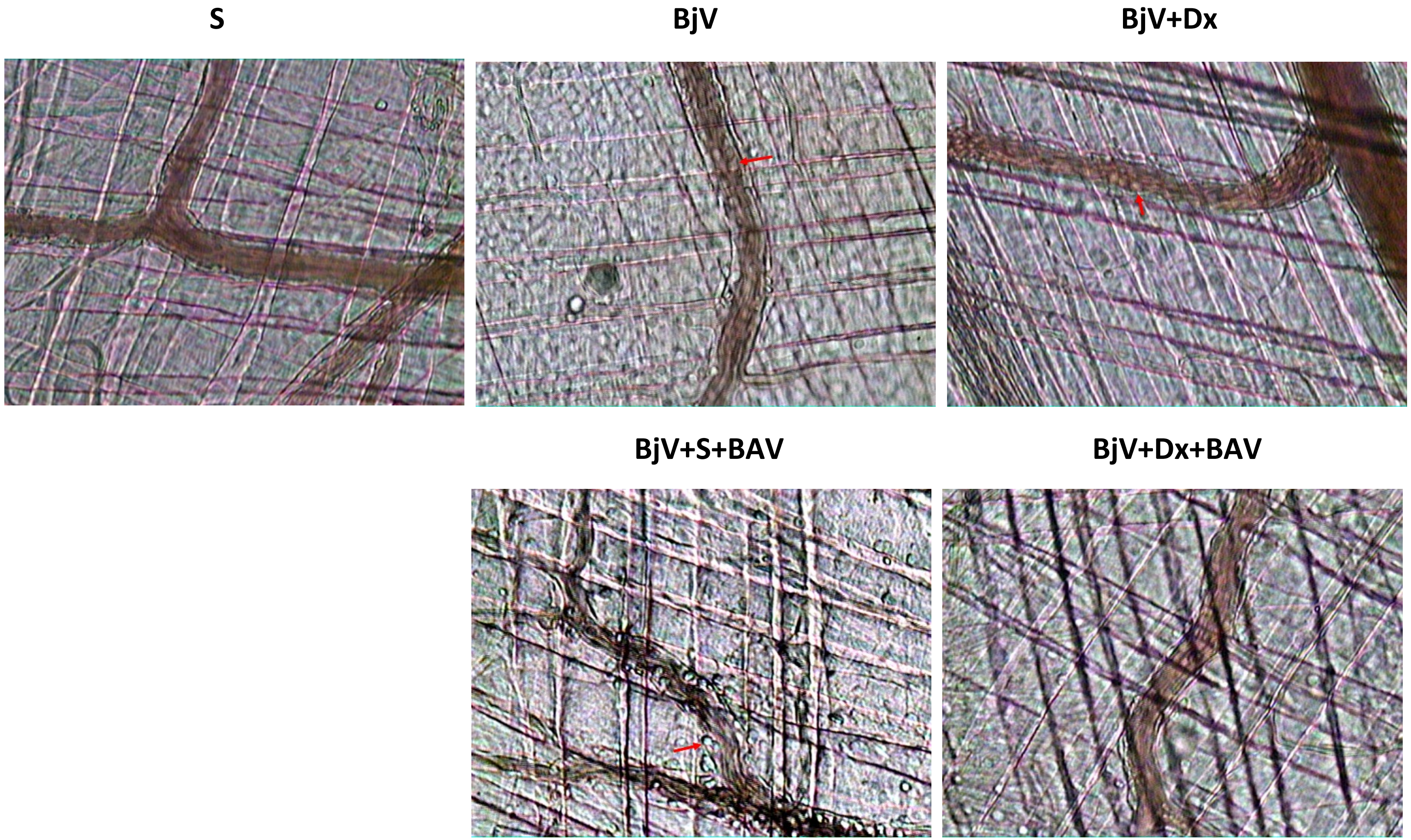

Supplementary Figure S1. The panel shows representative photomicrographs of the data of adhered cells of the Figure 4A. Images were obtained in a microscope, Axioplan II, Carl Zeiss, Germany, equipped with Achroplan objectives with 10.0 longitudinal distance, 0.25 numeric aperture, and 1.60 optovar. Red arrows indicate adhered leukocytes.

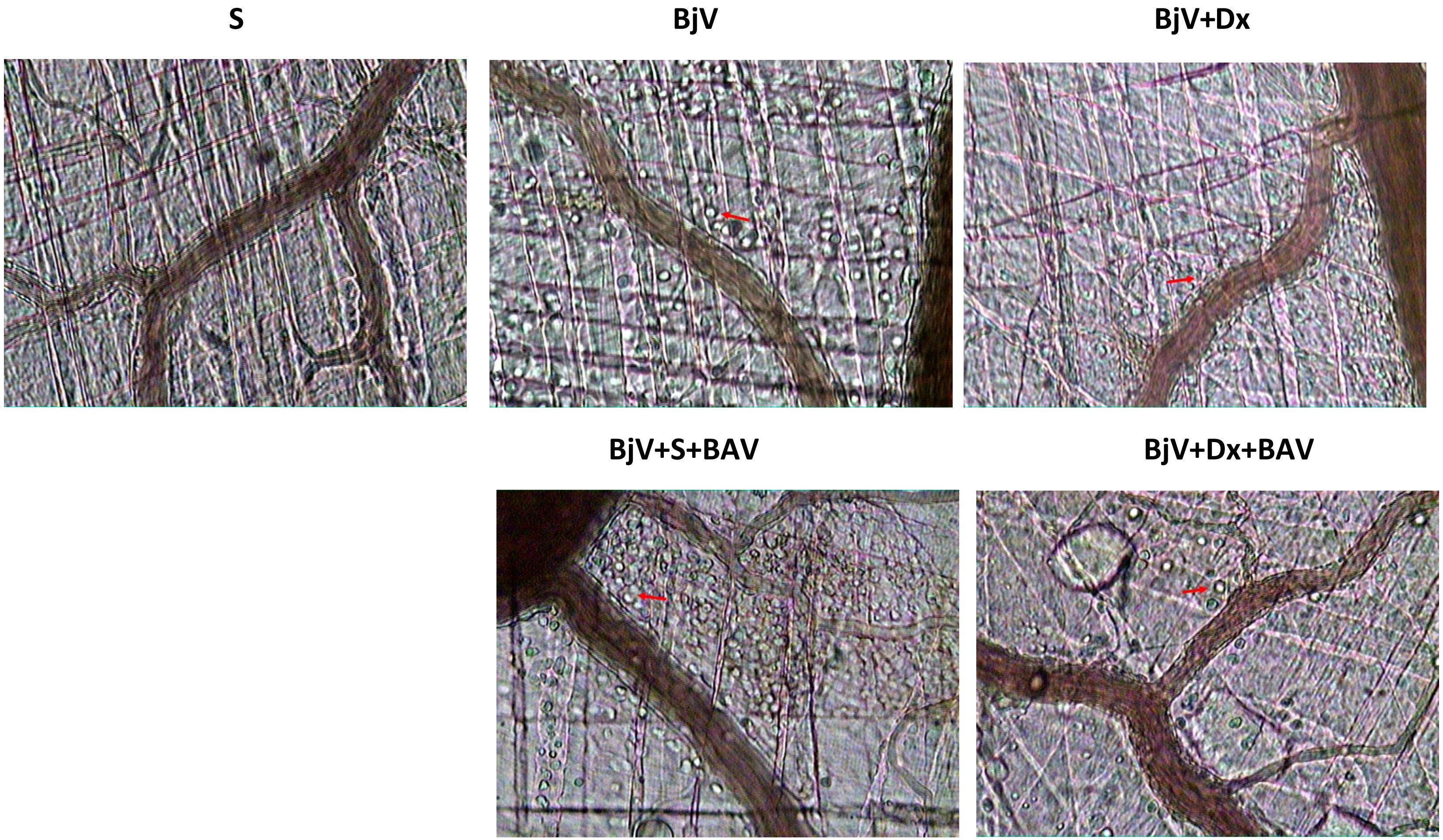

Supplementary Figure S2. The panel shows representative photomicrographs of the data of migrated cells of the Figure 4B. Images were obtained in a microscope, Axioplan II, Carl Zeiss, Germany, equipped with Achroplan objectives with 10.0 longitudinal distance, 0.25 numeric aperture, and 1.60 optovar. Red arrows indicate migrated leukocytes.
